# Development of a multiple cross displacement amplification combined with nanoparticles-based biosensor assay for rapid and sensitive detection of *Streptococcus pyogenes*

**DOI:** 10.1186/s12866-024-03189-5

**Published:** 2024-02-07

**Authors:** Zhiqian Dou, Ling Xie, Meiling Gao, Dexi Liu

**Affiliations:** 1 Department of Gynaecology and obstetrics, Taihe Hospital, Hubei University of Medicine, Shiyan, Hubei China; 2 Hubei Key Laboratory of Embryonic Stem Cell Research, Taihe Hospital, Hubei University of Medicine, Shiyan, China; 3 Department of Dermatology, Taihe Hospital, Hubei University of Medicine, Shiyan, Hubei China; 4 Department of Anesthesiology, Xiangyang No.1 People’s Hospital, Hubei University of Medicine, Xiangyang, Hubei China; 5 Department of Stomatology, Taihe Hospital, Hubei University of Medicine, Shiyan, Hubei China

**Keywords:** *Streptococcus pyogenes*, Multiple cross displacement amplification, Lateral flow biosensor, MCDA-LFB; Detection limit

## Abstract

**Background:**

*S. pyogenes*, is a primary pathogen that leads to pharyngitis and can also trigger severe conditions like necrotizing fasciitis and streptococcal toxic shock syndrome (STSS), often resulting in high mortality rates. Therefore, prompt identification and appropriate treatment of *S. pyogenes* infections are crucial in preventing the worsening of symptoms and alleviating the disease's impact.

**Results:**

In this study, a newly developed technique called multiple cross displacement amplification (MCDA) was employed to detect *S. pyogenes*,specifically targeting the *speB* gene, at a temperature of 63°C within 30 min. Then, an easily portable and user-friendly nanoparticles-based lateral flow biosensor (LFB) assay was introduced for the rapid analysis of MCDA products in just 2 min. The results indicated that the LFB offers greater objectivity compared to Malachite Green and is simpler than electrophoresis. The MCDA-LFB assay boasts a low detection limit of 200 fg and exhibits no cross-reaction with non-*S. pyogenes* strains. Among 230 clinical swab throat samples, the MCDA-LFB method identified 27 specimens as positive, demonstrating higher sensitivity compared to 23 samples detected positive by qPCR assay and 18 samples by culture. The only equipment needed for this assay is a portable dry block heater. Moreover, each MCDA-LFB test is cost-effective, priced at approximately $US 5.5.

**Conclusion:**

The MCDA-LFB assay emerges as a straightforward, specific, sensitive, portable, and user-friendly method for the rapid diagnosis of* S. pyogenes* in clinical samples.

**Supplementary Information:**

The online version contains supplementary material available at 10.1186/s12866-024-03189-5.

## Background

*Streptococcus pyogenes* (*S. pyogenes*), also known as Group A beta-hemolytic bacteria, is a significant Gram-positive pathogen linked to acute pharyngitis, accounting for 20 to 40% of cases in children and 5 to 15% of cases in adults [1–3]. While most cases of Streptococcal pharyngitis resolve spontaneously without antibiotics, a subset of patients faces potentially life-threatening conditions like necrotizing fasciitis and streptococcal toxic shock syndrome(STSS). In 2005, the global occurrence of Group A Streptococcus(GAS) disease was estimated to be as high as 18.1 million cases of severe disease, accompanied by 1.78 million new cases annually. This collective impact resulted in approximately 517,000 deaths each year [4]. According to the guidelines of the Infectious Diseases Society of America, *S. pyogenes* is susceptible to broad-spectrum antibiotics, with penicillin being the primary choice for treating *S. pyogenes* infections [5]. Hence, prompt diagnosis and appropriate treatment hold the key to managing symptoms, shortening disease duration, and alleviating the overall disease burden. However, the overlapping clinical presentations of streptococcal and viral pharyngitis often lead to diagnostic challenges, as clinicians relying solely on experience may struggle to provide accurate diagnoses prior to obtaining definitive throat culture results [6]. Consequently, 20–30% of patients are misdiagnosed, resulting in unnecessary antibiotic administration. This, in turn, can contribute to the development of antibiotic resistance and complicate the treatment of bacterial infections [7]. Therefore, there is a pressing need for a rapid diagnostic method for identifying *S. pyogenes* in modern clinical settings.

The conventional throat culture, which serves as the gold standard for detecting *S. pyogenes*, requires a lengthy 24–48 h waiting period.This delay poses challenges in promptly managing patients, particularly when symptoms are not pronounced. Several alternative rapid diagnostic methods are available, including Rapid Antigen Detection Test(RADT), PCR, qPCR and LAMP assays [8, 9]. However, the sensitivity of RADT (70–90%) and the need to retest negative results with culture add complexity to its use [10–13]. While PCR is highly accurate and often considered the reference standard, its reliance on specialized equipment and gel imaging hinders its widespread application. An independent qPCR system has emerged, but expense and potential non-target gene amplification are concerns. The LAMP method uses colorimetric indicators but relies on subjective visual assessment. Thus, a novel detection approach is crucial to overcome the limitations of existing methods.

The recently developed Multiple Cross Displacement Amplification(MCDA) assay, a novel nucleic acid amplification technique, operates at a consistent temperature range of 60 °C to 66 °C without requiring special equipment or extensive training [14]. Within the amplification mixture, a total of ten primers were meticulously designed to target the specific gene. These included displacement primers F1 and F2, cross primers CP1 and CP2, and amplification primers C1, C2, D1, D2, R1 and R2.

It's noteworthy that primers D1 and R1 were equipped with FITC (fluorescein) and biotin labels, respectively, at their 5' ends. These primers establish connections with the target gene or the newly formed strand, simultaneously initiating the polymerase extension to generate products of varying sizes. These encompass CP1/D1 products, double-labeled D1/R1 products, and other such variations. To facilitate detection, a highly convenient method employing gold nanoparticle-based lateral flow biosensors(LFB), which offer greater objectivity than traditional colorimetric indicators, has been used to identify MCDA products [15].

This study represents the pioneering utilization of nucleic acid amplification in conjunction with biosensors for the rapid and accurate detection of *S. pyogenes* through the *speB* gene. *speB*, an exotoxin gene encoding a potent cysteine proteinase, has been previously employed for the specific detection of *S. pyogenes* [8, 16]. In our study, we also chose to target the *speB* gene and developed an innovative isothermal amplification assay in combination with LFB, which shows greater sensitivity compared to the widely used LAMP assays [17]. Initially, we determined the optimal detection time and amplification temperature of the MCDA-LFB assays through a series of methods, including colorimetric indicators, LFB, and electrophoresis. Subsequently, the MCDA-LFB assay's sensitivity and specificity were meticulously assessed. Finally, we applied the MCDA-LFB assay to the detection of clinical specimens, and compared its performance with the reference methods of bacterial culture and qPCR.

## Materials and methods

### Reagents

The isothermal amplification kits, Disposable Lateral Flow biosensors(LFB), Malachite Green(MG) were all purchased from BeijingHaiTai-ZhengYuan Technology Co., Ltd.(Beijing). qPCR MasterMix reagents were purchased from Vazyme Biotech Co., Ltd.(Nanjing). DNA Microbiome Kits were purchased from Tiangen Biotech Co., Ltd.(Beijing).

### Preparation of a biosensor utilizing gold nanoparticles and a dipstick design

The dry-reagent strips were manufactured by following established procedures [15]. In summary, the assembly involved layering the sample pad, conjugate pad, NC membrane, and absorbent pad onto a plastic adhesive backing card. On the NC membrane, a solution containing the anti-FITC antibody (0.2 mg/mL) and biotin-BSA (2.5 mg/mL) conjugates was sprayed, giving rise to the test line (TL) and control line (CL), with a 5 mm separation between them. The conjugate pad of the strip received a deposition of SA-G within 0.01 M PBS (pH 7.4). Subsequently, the assembled cards were cut into 4-mm wide strips using equipment from Deli No. 8012. These finalized biosensors were meticulously packaged in a plastic container along with desiccant gel to regulate humidity and were stored at room temperature.

### Primer design

A set of MCDA primers targeting the *speB* gene of *S. pyogenes* was designed using PRIMER PREMIER 5.0 and PrimerExplorerV4(http://primerexplorer.jp/elamp4.0.0

**Fig. 1:**
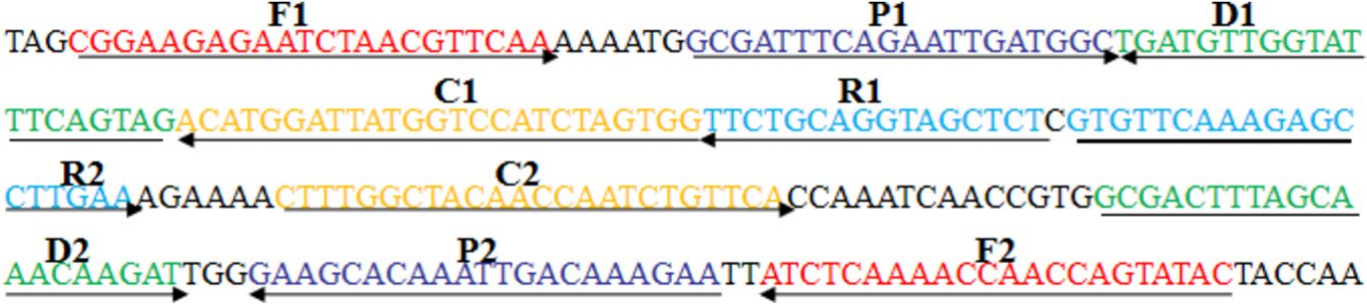
MCDA primer design for the *speB* gene

/index.html). The Integrated DNA Technologies design tool was used to analyze the hybrids and hairpin structures of the MCDA primers. All MCDA primers were blasted with the non-*S. pyogenes* genome to confirm their specificity for targeting the *speB* gene*.* The design of the MCDA primers is shown in Fig. 1 and their sequences are shown in Table 1. All MCDA primers were synthesized and purified by TsingKe Biotech Co., Ltd.(Beijing, China) at HPLC purification grade.

**Table 1:** The set of MCDA primers used in this study

Primers name	Sequences	Length	gene
speB-F1	5'-CGGAAGAGAATCTAACGTTCAA-3'	22 nt^*1^	speB
speB-F2	5'-GTATACTGGTTGGTTTTGAGAT-3'	22 nt	
speB-CP1	5'-CCACTAGATGGACCATAATCCATGTGCGATTTCAGAATTGATGGC-3'	45 mer ^*2^	
speB-CP2	5'-CTTTGGCTACAACCAATCTGTTCATTCTTTGTCAATTTGTGCTTC-3'	45 mer	
speB-C1	5'-CCACTAGATGGACCATAATCCATGT-3'	25 nt	
speB-C2	5'-CTTTGGCTACAACCAATCTGTTCA-3'	24 nt	
speB-D1	5'-CTACTGAAATACCAACATCA-3'	20 nt	
speB-D1^*3^	5'-FITC-CTACTGAAATACCAACATCA-3'	20 nt	
speB-D2	5'-GCGACTTTAGCAAACAAGAT-3'	20 nt	
speB-R1	5'-AGAGCTACCTGCAGAA-3'	16nt	
speB-R1^*4^	5'-Biotin-AGAGCTACCTGCAGAA-3'	16nt	
speB-R2	5'-GTGTTCAAAGAGCCTTGAA-3'	19 nt	

^1^ nt, nucleotide;

^2^ mer, monomeric;

^3^ D1*, 5ʹ-labeled with fluorescein (FITC);

^4^ R1*, 5ʹ-labeled with biotin;

### Bacterial strains and DNA extraction

In this study, a total of 24 bacterial strains were employed for the development of the MCDA-LFB assays (Table 2). To establish optimal reaction conditions and perform sensitivity analysis, we utilized the genome of the *S. pyogenes* strain ATCC19615 served as a positive control. In addition, the genomes of *S. pneumoniae* and *S. aureus* were employed as negative controls. To precisely determine the bacterial species, we conducted a thorough assessment by employing well-established culture techniques at the Department of Clinical Laboratory, Taihe Hospital of Hubei University of Medicine. Genomic DNA was extracted from the pure bacterial cultures using a DNA Mini Kit, and the concentration of extracted DNA was quantified via an ultraviolet spectrophotometer (Nano drop one, Thermo, Beijing, China) at A260/280.

**Table 2:** Strains used in this study and the results of MCDA assays

Bacteria	Strain no./source	No. of strains	MCDA-LFB result ^*1^
*Streptococcus pyogenes*	ATCC19615	1	+
	Isolated strains	5	+
*Streptococcus agalactiae*	Isolated strains	1	-
*Streptococcus pneumoniae*	Isolated strains	1	-
*Streptococcus mitis*	Isolated strain	1	-
*Streptococcus salivarius*	Isolated strain	1	-
*Streptococcus sanguinis*	Isolated strain	1	-
*Streptococcus dysgalactiae*	Isolated strain	1	-
*Streptococcus gordonii*	Isolated strain	1	-
*Streptococcus sinensis*	Isolated strain	1	-
*Streptococcus constellatus*	Isolated strain	1	-
*Streptococcus anginosus*	Isolated strain	1	-
*Enterococcus faecium*	Isolated strain	1	-
*Enterococcus raffinosus*	Isolated strain	1	-
*Staphylococcus epidermidis*	Isolated strain	1	-
*Staphylococcus aureus*	Isolated strain	1	-
*Pseudomonas aeruginosa*	Isolated strain	1	-
*Klebsiella pneumoniae*	Isolated strain	1	-
*Escherichia coli*	Isolated strain	1	-
*Candida albicans*	Isolated strain	1	-

^1^ + , positive; -, negative

### MCDA reactions

The *speB*-MCDA reactions were carried out in a 25 µL reaction mixture.This mixture included the following components: 0.4 µM of displacement primers F1 and F2, 0.8 µM of amplification primers C1, C2, D1*, D2, R1*, and R2, 1.6 µM of cross primers CP1 and CP2, 1 µL of Bst DNA polymerase(10U), 12.5 µL of 2*reaction buffer, 1.5 µL of Malachite Green, 7.8 µL of DW and 1 µL of DNA template.

In this study, we employed three distinct monitoring methods to verify *speB*-MCDA products: a colorimetric indicator (MG), gel electrophoresis and LFB. During the initial phase of preparing the MCDA reaction, MG was introduced.. As the amplification process advanced, the reaction temperature incrementally rose, leading to the separation of MG into two distinct components, denoted as Group A and Group B, resulting in the gradual loss of color within the reaction system. However, upon the generation of amplicons, Group A effectively bonded with the double-stranded amplification products, causing an immediate transition of the reaction system to a vibrant green color, serving as a clear indicator of a positive outcome. Following the completion of the amplification process, 5 µl of MCDA products were added to 2% gel electrophoresis, and run at 100 V for 20 min. Subsequently, the gel imaging system was employed to scrutinize the MCDA products, revealing a distinctive ladder-like band pattern indicative of *S. pyogenes* presence, while non*-S. pyogenes* samples displayed no discernible bands. When the LFB was utilized to detect MCDA products, both CL and TL are simultaneously visible for *S. pyogenes*, whereas CL is visible for non-*S. pyogenes*.

To confirm the effectiveness of the *speB*-MCDA primers, DNA from *S. pyogenes, S. pneumoniae*, *S. aureus*, and DW were introduced into the MCDA amplification reaction mixture. Subsequently, the MCDA products were subjected to analysis using the three aforementioned detection techniques.

We conducted amplification with 20 pg of DNA from the *S. pyogenes* strain ATCC19615, incrementally raising the temperature from 60 °C to 66 °C at 1 °C increments for 30 min. To pinpoint the most effective amplification temperature, we employed both MG and gel electrophoresis to identify the MCDA products. Furthermore, we employed DNA from the *S. pyogenes* strain ATCC19615,

spanning concentrations from 2 ng µL^−1^ to 2 fg µL^−1^, to ascertain the optimal detection time.

### Sensitivity of the LFB assay for detection of MCDA products

Serial tenfold dilutions of *S. pyogenes* ATCC19615 genomic DNA, ranging from 2 ng µL^−1^ to 2 fg µL^−1^, were added to each *speB*-MCDA reaction(1.0 µL each). The minimum amount of DNA that could be reliably detected by LFB was determined as the limit of detection. At the same time, agarose gel electrophoresis and a colorimetric indicator were used to further confirm the LFB detection results for MCDA products.

### Specificity of the LFB assay for detection of MCDA products

A total of 24 strains, including *Streptococcus* species of *S. pyogenes*, *S. agalactiae*, *S. pneumoniae*, *S. mitis*, *S. salivarius*, *S. sanguinis*, *S. dysgalactiae*, *S. gordonii*, *S. sinensis*, *S. constellatus*, *S. anginosus*, *E. faecalis* and *E. faecium*, and non*-Streptococcus* species of *S. aureus*, *S. epidermidis*, *K. pneumoniae*, *P. aeruginosa*, *E. coli*, *C. albicans*, were used to evaluate the specificity of the *S. pyogenes* MCDA-LFB assays.

### Validation of MCDA-LFB assay on clinical throat swabs

A total of 230 clinical throat swab specimens were collected from subjects aged > 2 years with signs and symptoms of pharyngitis at the Paediatrics Outpatient Department of Taihe Hospital(Shiyan, Hubei, China) between April and May. Two throat swabs were collected from each patient: one for the reference method of culture technique and another one for the MCDA-LFB assay and qPCR assay. The qPCR primer sequences used in this study were as follows: speB forward5'-CGGAAGA.

GAATCTAACGTTCAA-3', and speB reverse 5'-GTATACTGGTTGGTTTTGAG.

AT-3'. The qPCR reaction system comprises the following components: 0.4 µM speB forward primer, 0.4 µM speB reverse primer, 10 µL 2*TB Green Fast qPCR Mix, 6.4 µL DW, and 2 µL DNA. The qPCR reaction conditions are as follows: 95 °C for 30 s, followed by 40 cycles of denaturation at 95 °C for 5 s, and annealing/extension at 60 °C for 34 s.

## Results

### Confirmation and analysis of *speB*-MCDA products

Initially, the DNA samples were categorized into two distinct groups: a positive group(*S. pyogenes*) and a negative group (*S. pneumoniae and S. aureus*). Subsequently, both the positive and negative DNA groups, along with the blank group(DW), were individually introduced into separate MCDA reactions to assess the effectiveness of the *speB*-MCDA primers. Following a 60 min amplification at 61 °C, we employed three monitoring techniques to assess MCDA products: MG, agarose gel.

**Fig. 2:**
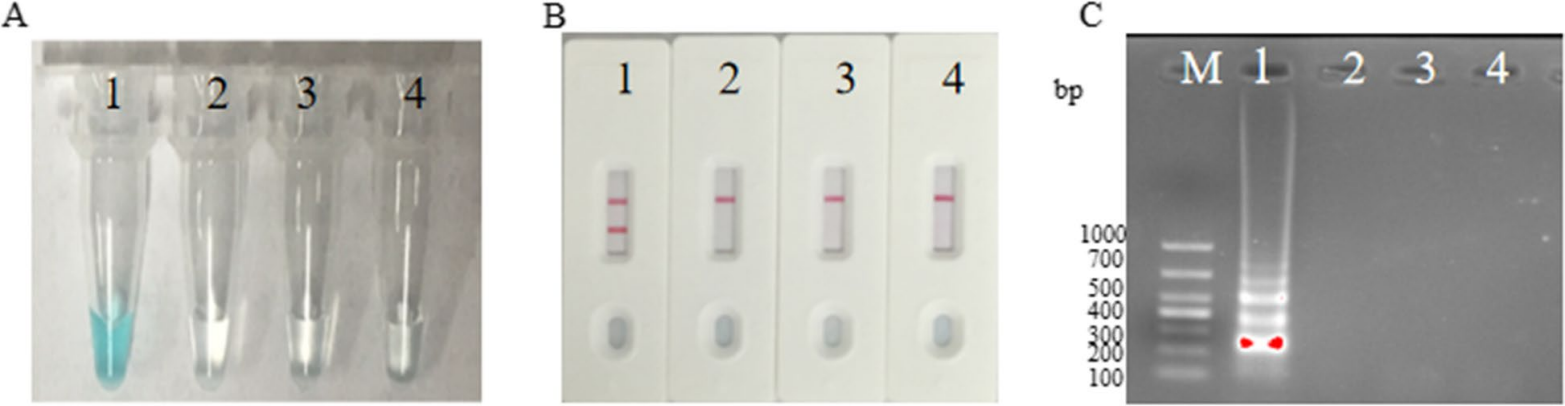
Detection of *S. pyogenes*-MCDA products Three methods were used to analyse MCDA amplicons, including Malachite Green (**A**), lateral flow biosensor (**B**) and 2% Agarose gel electrophoresis (**C**). Lane M, DNA Maker DL1000. 1, positive group(*S. pyogenes* strain); 2, negative group (*S. pneumoniae*); 3, negative group (*S. aureus*); 4, blank group (DW).TL, test line.CL, control line

Electrophoresis and LFB. As observed in Fig. 2, the reaction mixture exhibited a vibrant green coloration upon the completion of amplification when using DNA from the positive group*.* In contrast, it remained colorless when employing DNA from the negative and blank groups*.* Using the LFB, we detected the presence of two red lines, one at the CL position and the other at the TL position, in the case of *speB*-MCDA products. Conversely, for both the negative and blank groups, only a single red line materialized at the CL position. By 2% gel electrophoresis, a ladder band was visible for the positive group*,* but not for the negative and blank groups. These results demonstrated that the set of *speB*-MCDA primers was a good candidate for the establishment of the MCDA-LFB approach.

### Optimal amplification temperature evaluated for the *speB*-MCDA primers

**Fig. 3:**
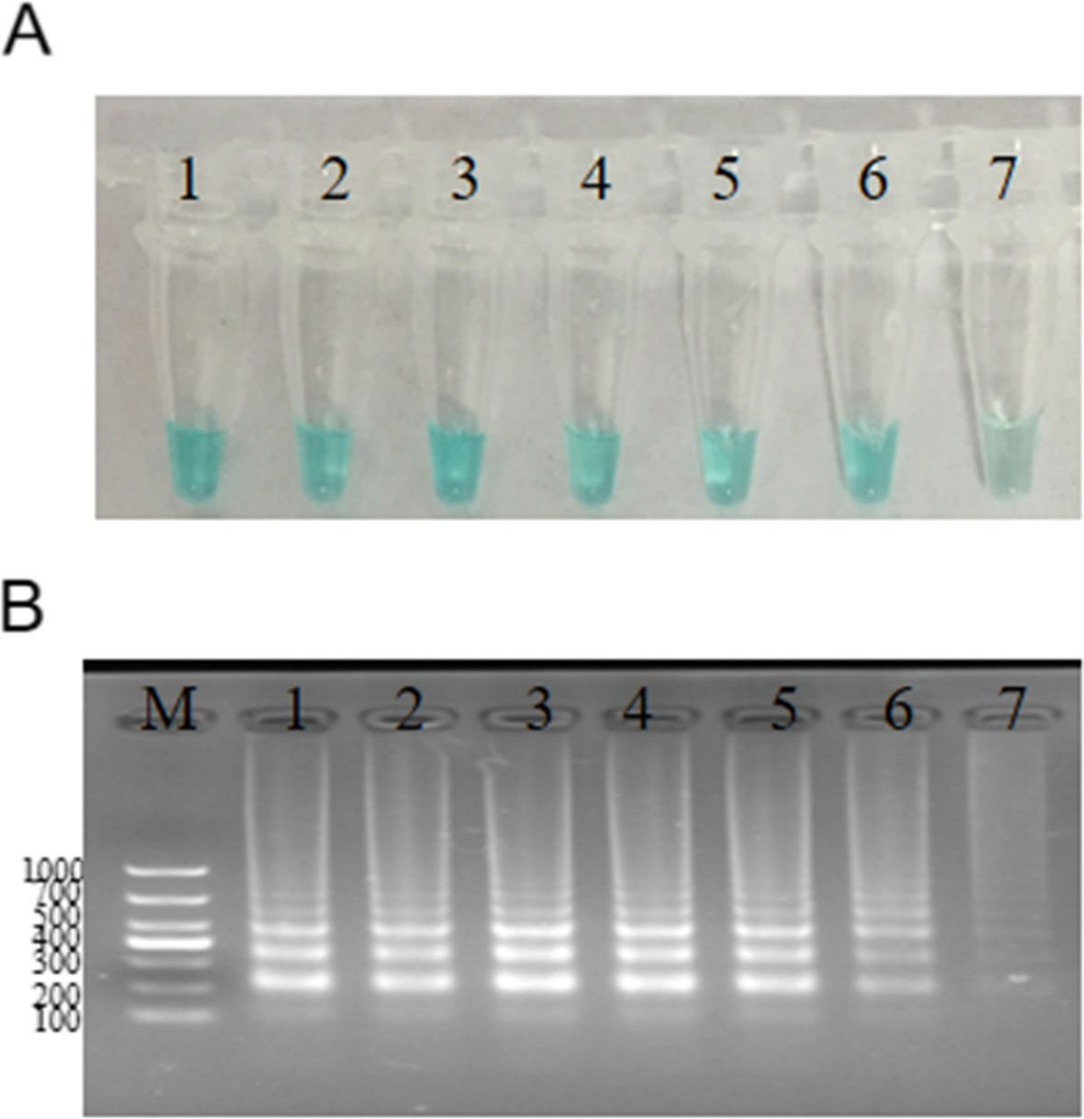
Optimization of amplification temperature for *S. pyogenes*-MCDA primers 20 pg of *S. pyogenes* genome added to the MCDA reaction was incubated in a range between 60 °C and 66 °C for 30 min. Two methods, including MG and agarose gel electrophoresis, were then used to evaluate the amplification efficiency of the *S. pyogenes-*MCDA assays. Tubes/agarose gel electrophoresis 1–7 represent 60 °C, 61 °C, 62 °C, 63 °C, 64 °C, 65 °C, 66 °C respectively

To determine the optimal amplification temperature for the primer set, we conducted the MCDA reaction at seven distinct temperatures, ranging from 60 °C to 66 °C in 1 °C increments, using 20 pg µL^−1^ DNA for 30 min. We evaluated the amplification efficiency of the primers using two diagnostic methods: MG and agarose gel electrophoresis. As observed in Fig. 3, the MCDA reaction carried out within the temperature range of 60–65 °C exhibited a more intense green coloration when evaluated with MG compared to the reaction conducted at 66 °C. In the case of amplicon analysis using agarose gel electrophoresis, no substantial disparities in amplification were observed across the temperature range of 60 °C to 64 °C. However, there was a notable reduction in the brightness of the target bands at 65 °C and 66 °C. Consequently, we opted for a temperature of 63 °C as the ideal choice for subsequent experiments.

### Sensitivity of *speB*-MCDA-LFB assays

**Fig. 4:**
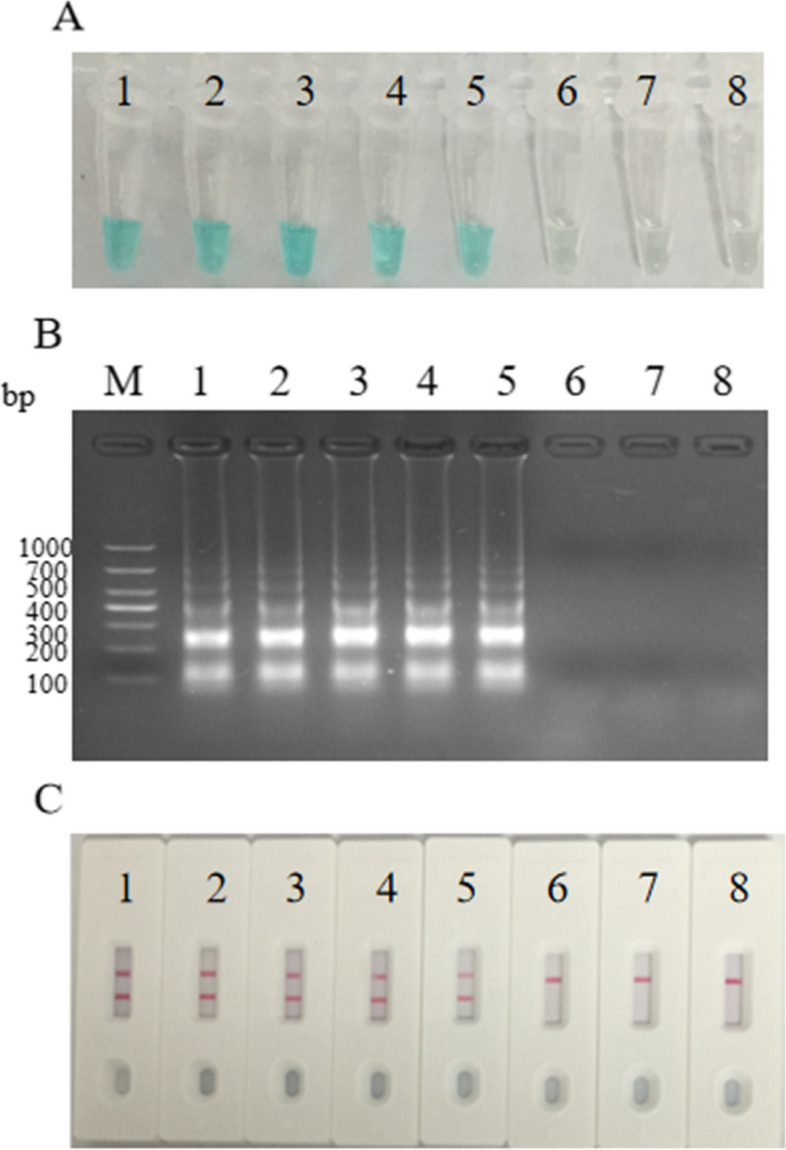
Sensitivity of the LFB assay for *S. pyogenes-*MCDA products Measurement techniques of colorimetric indicator (**A**), gel electrophoresis (**B**) and lateral flow biosensor (**C**) were used to analyze MCDA amplicons. A tenfold serial dilutions of the template in a range between 2 ng and 2 fg were prepared for evaluating the detection line.Tubes (A)/Lanes (B)/Biosensors (C) 1–8 respectively represent *S. pyogenes* strain ATCC19615 DNA levels of 2 ng, 200 pg, 20 pg, 2 pg, 200 fg, 20 fg and 2 fg per reaction, and a blank control (DW)

We determined the minimum detection threshold of the MCDA-LFB assay for *S. pyogenes* by conducting serial tenfold dilutions of DNA within the MCDA reaction. As observed in Fig. 4, it became evident that only at an initial DNA concentration of 200 fg µL^−1^ and higher did the MCDA reactions yield positive results, marked by the appearance of two red lines(CL and TL) on the LFB. Furthermore, the MG assay and agarose gel electrophoresis assay also demonstrated the ability to detect *speB-*MCDA products from an initial DNA level of 200 fg.

### Time optimization of the MCDA-LFB assay

**Fig. 5:**
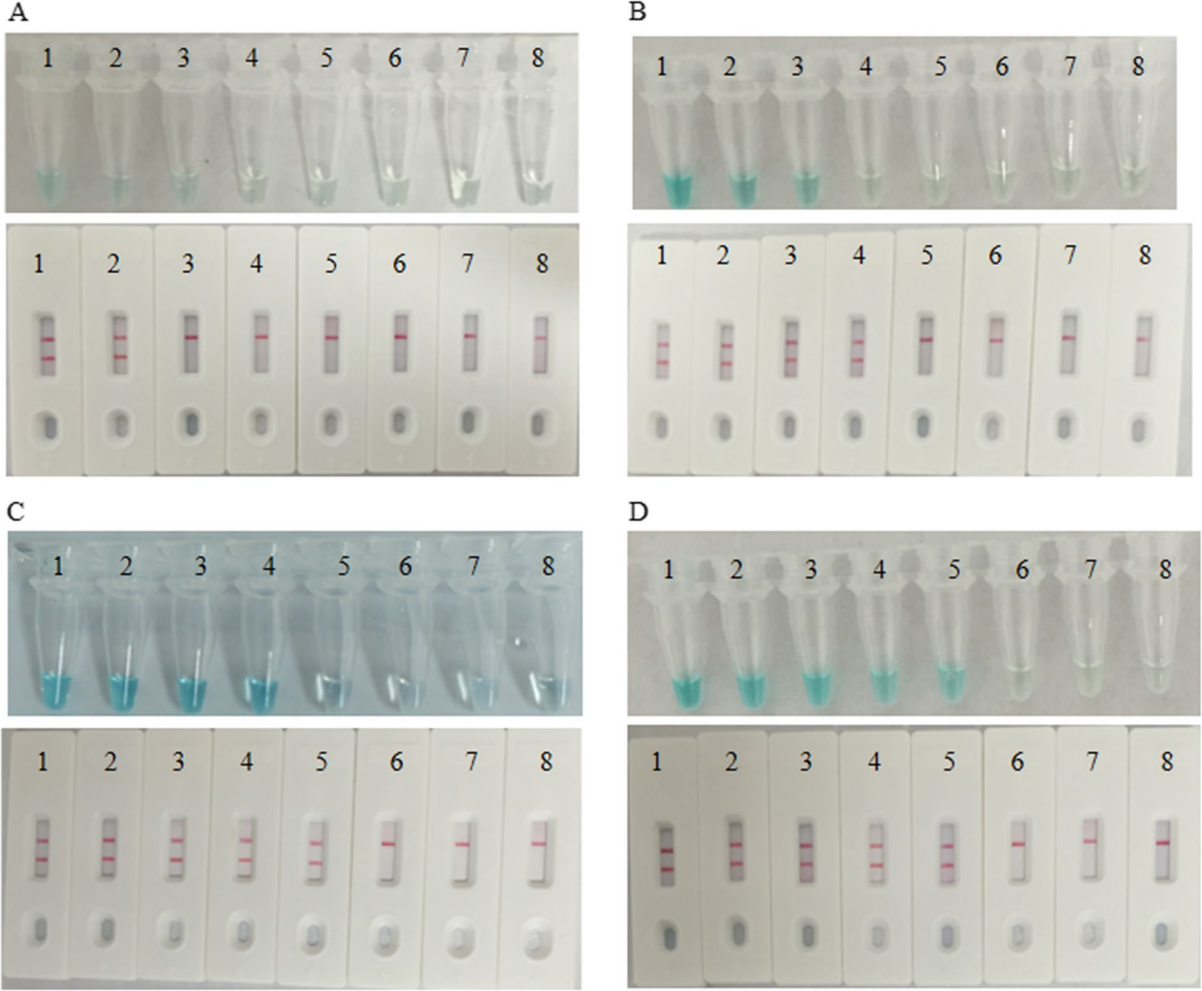
Optimal detection time for the *S. pyogenes* MCDA-LFB assay. Four reaction times (**A**, 10 min; **B**, 20 min; **C**, 30 min; **D**, 40 min) were selected to determine the optimal detection time at 63 °C. Tubes/biosensors 1- 8 represent *S. pyogenes* strain ATCC19615 DNA levels of 2 ng, 200 pg, 20 pg, 2 pg, 200 fg, 20 fg and 2 fg per reaction, and a blank control (DW), respectively. The minimum detection limit was observed when MCDA lasted for 30 min (C)

The optimal time was determined using DNA extracted from *S. pyogenes* at concentrations ranging from 2 ng µL^−1^ to 2 fg µL^−1^. As shown in Fig. 5, when the MCDA reaction was incubated at 63 °C for 10 min, it was observed that an initial DNA quantity of either 2 ng or 200 pg within the MCDA reactions could generate duplex amplicons within the MCDA reactions. This led to the appearance of red lines at both the CL and TL positions on the LFB. Using the MG assay, it is challenging to definitively determine the occurrence of amplification, primarily because the reaction mixture's color remained nearly colorless when the initial DNA amount was below 200 pg. By extending the amplification time to 20 min, the LFB becomes capable of detecting MCDA products even when the initial DNA amount is 2 pg or more. Nevertheless, the MG assay encounters a similar issue as it did at 10 min, with the reaction appearing nearly colorless when the initial DNA amount is 2 pg or less. When we extend the amplification time to 30 min and 40 min, the LFB becomes capable of detecting MCDA products when the initial DNA amount is 200 fg or more. However, it should be noted that the MG assay cannot detect positive samples at 30 min but can do so at 40 min. Consequently, we determined that a 30-min amplification time is the optimal choice for the MCDA-LFB assay.

### Analytical specificity of the MCDA-LFB assay

**Fig. 6:**
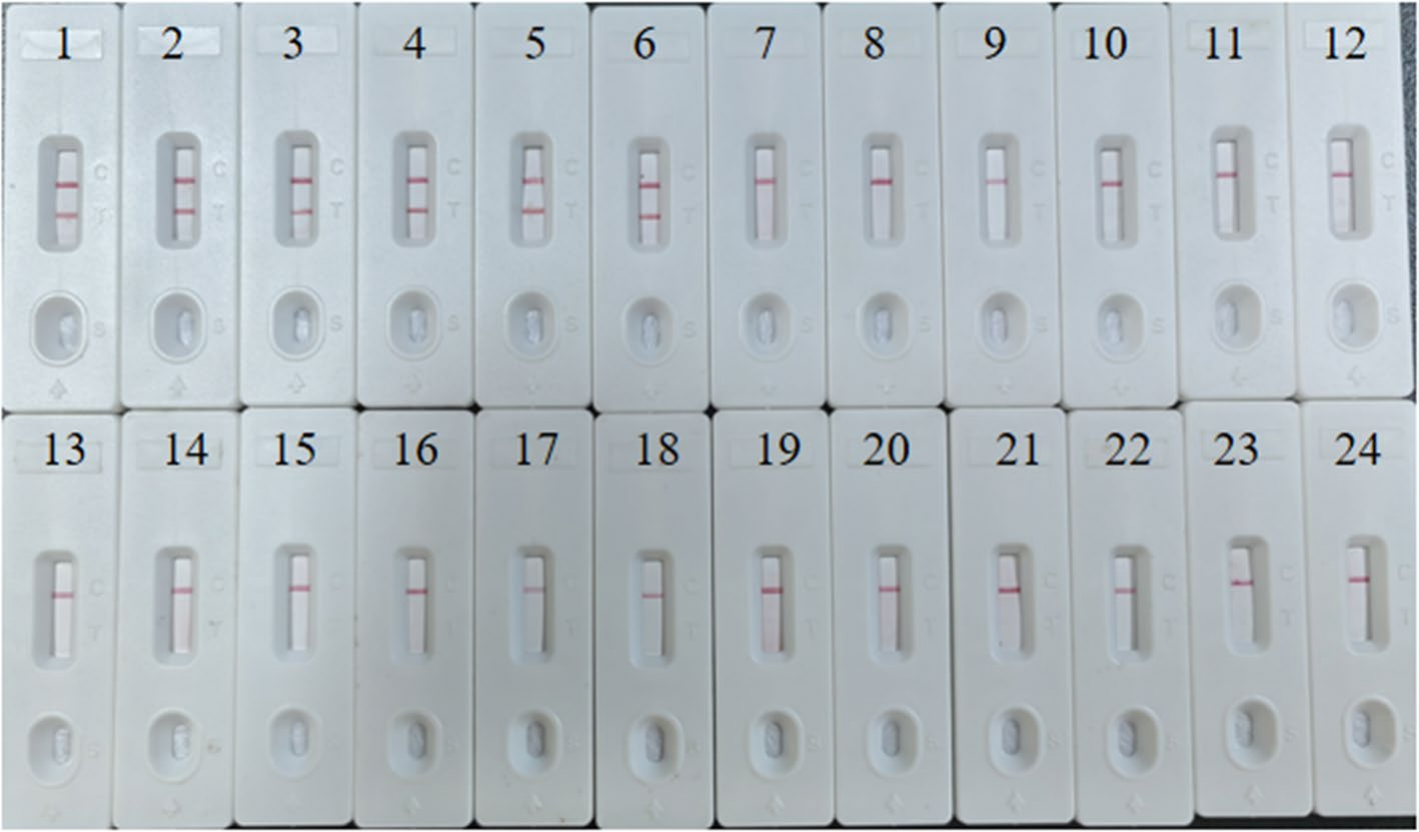
Specificity of *S. pyogenes*-MCDA-LFB assays 24 strains were used to evaluate the specificity of the MCDA-LFB assay. TL and CL were visible in LFB for all *S. pyogenes,* and only CL was observed for non*-S. pyogenes*. 1, Positive control (1, *Streptococcus pyogenes* strain ATCC19615); 2–6, *Streptococcus pyogenes*; 7, *Streptococcus agalactiae*. 8, *Streptococcus pneumoniae*; 9, *Streptococcus mitis*; 10, *Streptococcus salivarius*; 11, *Streptococcus sanguinis*; 12, *Streptococcus dysgalactiae*; 13, *Streptococcus gordonii*; 14, *Streptococcus sinesis*; 15, *Streptococcus constellatus*; 16, *Streptococcus anginosus*; 17, *Enterococcus faecium*; 18, *Enterococcus raffinosus*; 19, *Staphylococcus aureus*; 20, *Staphylococcus epidermidis*; 21, *Pseudomonas aeruginosa*; 22, *Klebsiella pneumoniae*; 23, *Escherichia coli*; 24, *Candida albicans*;

Under the optimal reaction conditions, we assessed the specificity of the *S. pyogenes* MCDA-LFB assay by testing it against a panel of 6 *S. pyogenes* strains and 18 non-*S. pyogenes* strains, as shown in Table 2. The outcomes, as observed in Fig. 6, revealed that only *S. pyogenes* strains generated amplicons, resulting in the appearance of two red lines on the LFB. In contrast, none of the 18 non-*S. pyogenes* strains generated any test lines on the LFB. These results conclusively demonstrate the absence of cross-reactivity with non-*S. pyogenes* strains and confirm the specificity of the primer set.

### Validation of the MCDA-LFB assay on clinical throat swabs

**Fig. 7:**
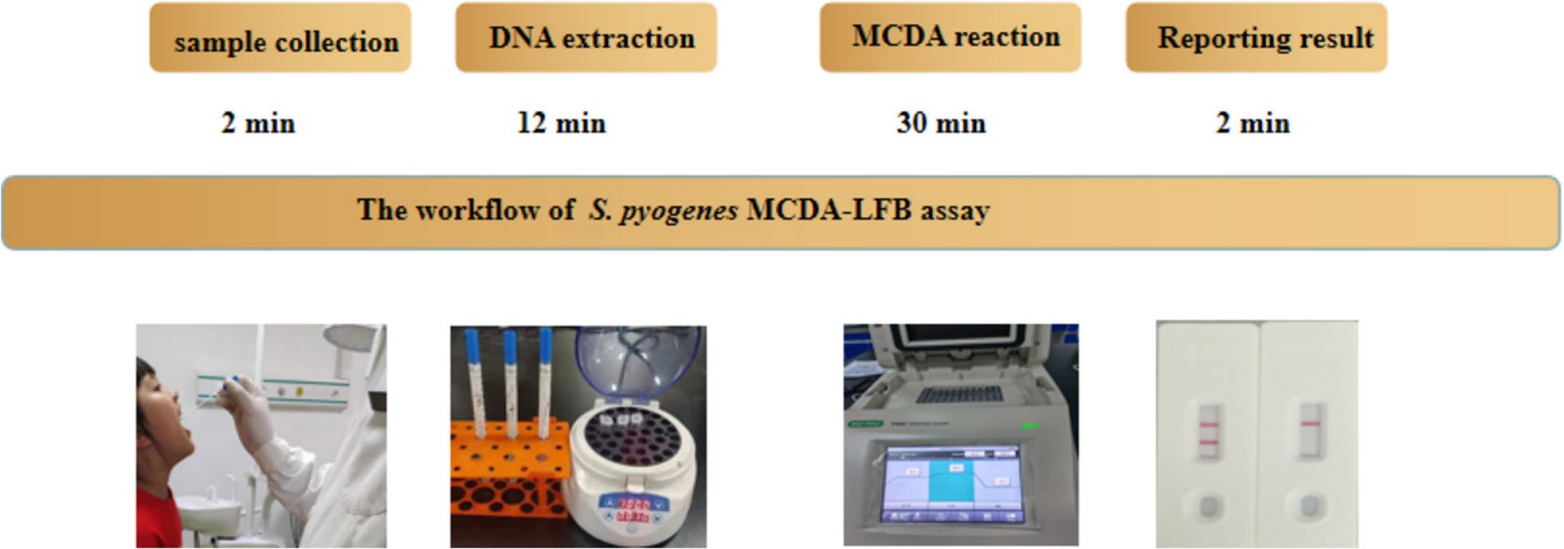
The workflow of *S. pyogenes* MCDA-LFB assays The whole process of *S. pyogenes* MCDA-LFB test takes about 46 min, including sample collection (2 min), DNA extraction (12 min), MCDA reaction (30 min) and result reporting (2 min)

To assess the viability of the MCDA-LFB assays when applied to clinical specimens, a comprehensive evaluation was conducted involving 230 clinical throat swab specimens. Concurrently, these samples were subjected to analysis using both the qPCR assay and culture methods. The results unveiled that among the examined specimens, 27 were identified as positive by the MCDA-LFB method, while 23 were identified as positive by the qPCR assay and 18 were identified as positive by culture (Table 3). In comparison to the culture-based assay, the MCDA-LFB method demonstrated a sensitivity of 100%, a specificity of 95.9%, a positive predictive value of 66.7%, and a negative predictive value of 100%. When compared to the qPCR assay, the MCDA-LFB assay exhibited a sensitivity of 100%, a specificity of 98.1%, a positive predictive value of 85.2%, and a negative predictive value of 100%. The complete process for obtaining an MCDA-LFB result from a clinical specimen takes approximately 46 min. This duration encompasses several steps: 2 min for sample collection, 12 min for DNA extraction, 30 min for the amplification process, and 2 min for the LFB analysis. Notably, this timeframe is significantly shorter than the 24–48 h required for culture and the 84 min for a qPCR assay. As depicted in Fig. 7, the graphical diagram illustrates the process from sample collection to the final result assessment in the MCDA-LFB assay.

**Table 3:** The MCDA-LFB assay compared to culture and qPCR in detection *Streptococcus pyogenes* from throat swab

MCDA-LFB	Culture		qPCR	
	Positive	Negative	Positive	Negative
Positive	18	9	23	4
Negative	0	213	0	213

## Discussion

*S. pyogenes* gives rise to a spectrum of disease ranging from mild streptococcal pharyngitis to severe conditions such as necrotizing fasciitis and streptococcal toxic shock syndrome(STSS), contributing to substantial morbidity and mortality worldwide [18]. With an estimated annual toll of 620 million mild cases and 517,000 deaths, prompt and precise diagnosis becomes paramount for effective management. While the mean case fatality rate is only at 8–16%, delayed treatment can escalate invasive GAS mortality to 60–70% [19]. Conventional culture-based assays, which rely on intricate equipment and skilled personnel, are inadequate for modern clinical demands. RADT provide swift results without complex setup but lack sensitivity and often require culture confirmation for negative results. Hence, an advanced detection method with rapidity, heightened sensitivity, and convenient qualitative analysis is imperative. Here, we present MCDA coupled to a biosensor that has been successfully used for the detection of *Legionella pneumophila* and *Pseudomonas aeruginosa* [20, 21]. The *S. pyogenes*-MCDA-LFB assay exhibited remarkable sensitivity, detecting as low as 200 fg, a 7.45-fold enhancement over the *S. pyogenes* LAMP assay with a detection threshold of 1.49 pg [9].

The widespread conservation of *speB* across *S. pyogenes* strains led us to choose it as our target gene. Our results highlight the discriminatory power of the *S. pyogenes*-MCDA-LFB assay, distinguishing it from 18 other species, underscoring its excellent specificity.

Figure 5A, with the MG method, the level of amplification is not substantial enough to produce noticeable color distinctions, highlighting a limitation of the MG technique. The sensitivity of the LFB is equivalent to that of the reference method using agarose gel. Therefore, when the LFB successfully detects the MCDA products, the MG may not be able to detect them. As a result, the results obtained from the MG were considered false-negative outcomes.

To further evaluate the clinical applicability of the MCDA-LFB assay, we analyzed 230 throat swab samples using three methods, including culture, qPCR, and MCDA-LFB assay. For the MCDA-LFB assay and qPCR assays, DNA extraction wasn't needed as the reactions could be performed directly using boiled samples.The distinct positive outcomes yielded by the three methods underscore the heightened sensitivity of the MCDA-LFB assay in comparison to qPCR and culture. In addition, the MCDA-LFB assay's performance exhibited minimal susceptibility to variations in throat sample composition. Remarkably swift, the entire process of the MCDA-LFB assay, from specimen collection to result interpretation, is completed in approximately 46 min, signifying its rapidity. These attributes collectively not only streamline intricate procedural steps but also lead to reduced time and cost investments. While the present study showcased the MCDA-LFB assay's exceptional sensitivity and specificity, further validation through a broader range of clinical specimens is warranted to solidify its clinical applicability.

In addition, the LFB was employed for MCDA product analysis, offering advantages over agar gel electrophoresis, fluorescent indicators and real-time turbidimeters. Gel electrophoresis is time-consuming and requires additional gel imaging equipment. When only a few templates were available, it was challenging to distinguish between the fluorescence indicator colors of positive and negative specimens, resulting in inaccurate negative assessments. The real-time turbidimeter is a costly device that cannot be used in the resource-limited settings. In contrast, the LFB is a portable solution that can be stored at room temperature, and weighs only 4.3 g, eliminating the need for specialized thermal equipment. The sole equipment required in the whole process is a thermostat or a water bath, with a cost of $US 228. The reagent cost for the MCDA-LFB test amounts to $US 5.5, comprising $US 3 for the MCDA per reaction and $US 2 for the LFB. Since the entire procedure is straightforward, no special training is required. The sole drawback associated with the LFB pertains to the need for opening the lid during detection, thereby increasing the potential for aerosol pollution. However, this form of contamination can be effectively prevented by incorporating the AUDG enzyme into the MCDA reaction mixture before the amplification process begins. All these features showed that the LFB is objective, convenient, simple, economical and easy-to-use.

## Conclusion

We have successfully developed a portable and user-friendly MCDA-LFB assay for the rapid detection of *S. pyogenes* in clinical throat swab samples, with a total assay time of approximately 46 min. This simplified process eliminates the need for specialized thermal cyclers by utilizing a simple thermostat. The MCDA-LFB assay demonstrated superior detectability compared to culture and qPCR methods. With these significant advantages, it holds great potential as a diagnostic tool for the swift and accurate detection of *S. pyogenes*, offering high sensitivity and specificity. Further collection of clinical specimens is necessary to comprehensively assess the clinical applicability of the MCDA-LFB assay.

### Supplementary Information

**Additional file 1.**

## Data Availability

All data analysed during this study are included in this manuscript.
