# Supplementary material for: Development of a multiple cross displacement amplification combined with nanoparticles-based biosensor assay for rapid and sensitive detection of Streptococcus pyogenes
Source: BMC Microbiol. 2024 Feb 7;24:51. doi: 10.1186/s12866-024-03189-5 (PMC10848541; doi:10.1186/s12866-024-03189-5)

**The original agarosegel of Fig.2C in the manuscript**

Agarose gel electrophoresis. Lane M, DNA maker DL1000. 1, positive group(*S. pyogenes* strain); 2, negative group (*S. pneumoniae* ); 3, negative group (*S. aureu*); 4, blank group (DW).TL, test line.CL, control line.


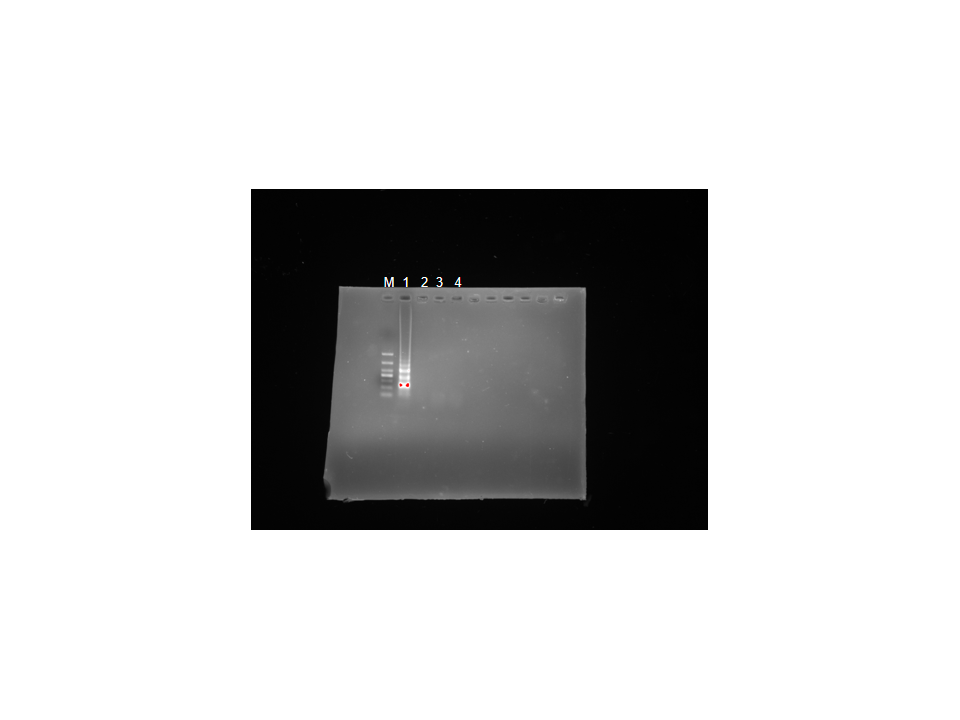


**The original agarose gel of Fig.3B in the manuscript**

20 pg *S. pyogenes* genome added to MCDA reaction were incubated in a range bettween 60°C and 66°C in 30 min. Then, Agarose gel electrophoresis was used to evaluate the amplification efficiency of *S. pyogenes-*MCDA assays. Agarose gel electrophoresis 1-7 respectively represent 60 °C, 61 °C, 62 °C, 63 °C, 64 °C, 65 °C, 66 °C.


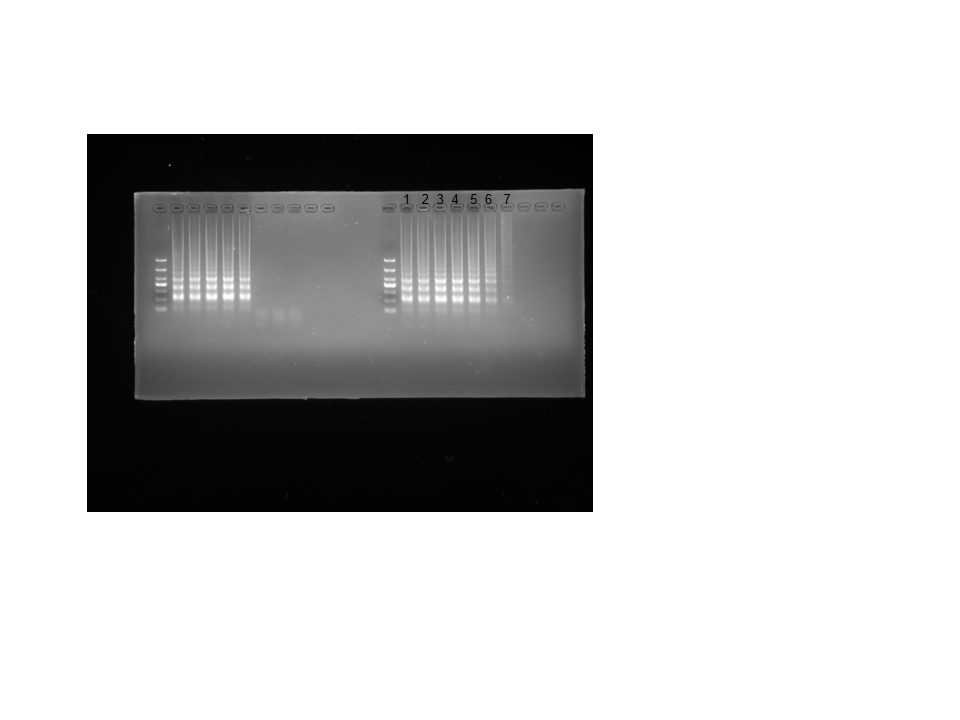


**The original agarose gel of Fig.4B in the manuscript**

Gel electrophoresis was used to analyze MCDA amplicons. A 10-fold serial dilutions of the template in a range between 2 ng and 2 fg were prepared for evaluating the detection line.Lanes 1–8 respectively represent *S. pyogenes* strain ATCC19615 DNA levels of 2 ng, 200 pg, 20 pg, 2 pg, 200 fg, 20 fg and 2 fg per reaction, and a blank control (DW).


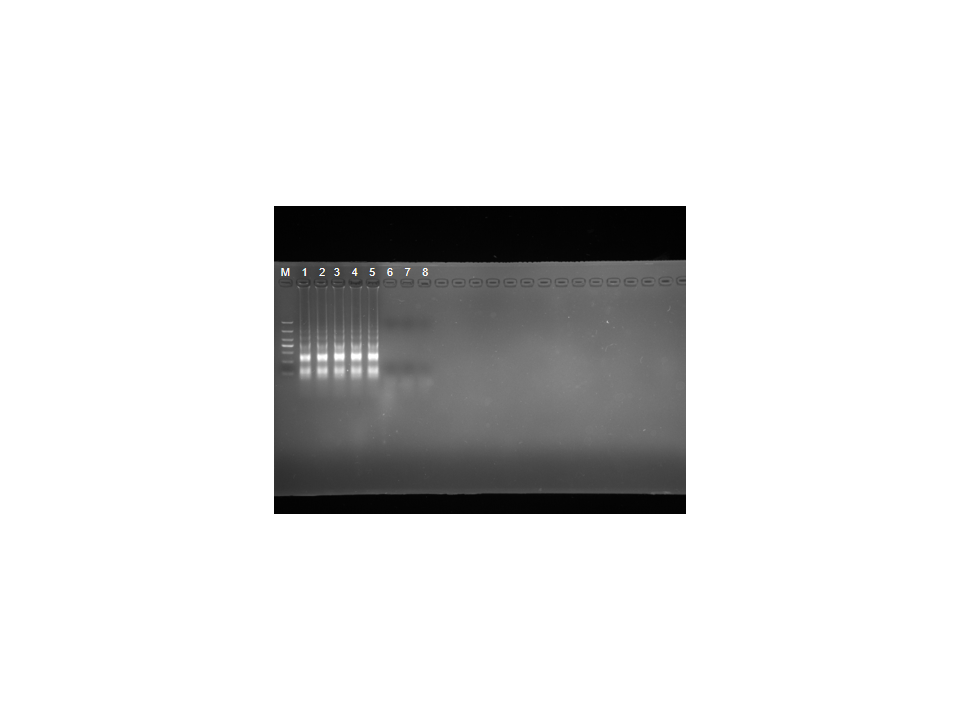

Supplement: Supplementary file 1 — Additional file 1. [file 12866_2024_3189_MOESM1_ESM.doc]
